# Topographical and Compositional Gradient Tubular Scaffold for Bone to Tendon Interface Regeneration

**DOI:** 10.3390/pharmaceutics14102153

**Published:** 2022-10-10

**Authors:** Eleonora Bianchi, Angela Faccendini, Elena Del Favero, Caterina Ricci, Laura Caliogna, Barbara Vigani, Francesco Claudio Pavesi, Cesare Perotti, Rui M. A. Domingues, Manuela E. Gomes, Silvia Rossi, Giuseppina Sandri

**Affiliations:** 1 Department of Drug Sciences, University of Pavia, 27100 Pavia, Italy; 2 Department of Medical Biotechnology and Translational Medicine, University of Milan, LITA, 20090 Segrate, Italy; 3 Orthopedy, Fondazione IRCCS Policlinico San Matteo, 27100 Pavia, Italy; 4 Immunohaematology and Transfusion Service, Apheresis and Cell Therapy Unit, Fondazione IRCCS Policlinico S. Matteo, 27100 Pavia, Italy; 5 3B’s Research Group, i3Bs—Research Institute on Biomaterials Biodegradables and Biomimetics, University of Minho, 4805-017 Guimarães, Portugal

**Keywords:** electrospun nanofibers, chitosan, pullulan, chondroitin sulfate, hydroxyapatite nanoparticles, stem cells differentiation, tendon tissue engineering

## Abstract

The enthesis is an extremely specific region, localized at the tendon–bone interface (TBI) and made of a hybrid connection of fibrocartilage with minerals. The direct type of enthesis tissue is commonly subjected to full laceration, due to the stiffness gradient between the soft tissues and hard bone, and this often reoccurs after surgical reconstruction. For this purpose, the present work aimed to design and develop a tubular scaffold based on pullulan (PU) and chitosan (CH) and intended to enhance enthesis repair. The scaffold was designed with a topographical gradient of nanofibers, from random to aligned, and hydroxyapatite (HAP) nanoparticles along the tubular length. In particular, one part of the tubular scaffold was characterized by a structure similar to bone hard tissue, with a random mineralized fiber arrangement; while the other part was characterized by aligned fibers, without HAP doping. The tubular shape of the scaffold was also designed to be extemporarily loaded with chondroitin sulfate (CS), a glycosaminoglycan effective in wound healing, before the surgery. Micro CT analysis revealed that the scaffold was characterized by a continuous gradient, without interruptions from one end to the other. The gradient of the fiber arrangement was observed using SEM analysis, and it was still possible to observe the gradient when the scaffold had been hydrated for 6 days. In vitro studies demonstrated that human adipose stem cells (hASC) were able to grow and differentiate onto the scaffold, expressing the typical ECM production for tendon in the aligned zone, or bone tissue in the random mineralized part. CS resulted in a synergistic effect, favoring cell adhesion/proliferation on the scaffold surface. These results suggest that this tubular scaffold loaded with CS could be a powerful tool to support enthesis repair upon surgery.

## 1. Introduction

The enthesis is a highly specific region localized at the tendon–bone interface (TBI). In this type of hybrid tissue, the fibrocartilage derived from the tendon part, is gradually reinforced with hydroxyapatite from bone, which renders it increasingly hard, since it becomes bone tissue.

**Figure 1:**
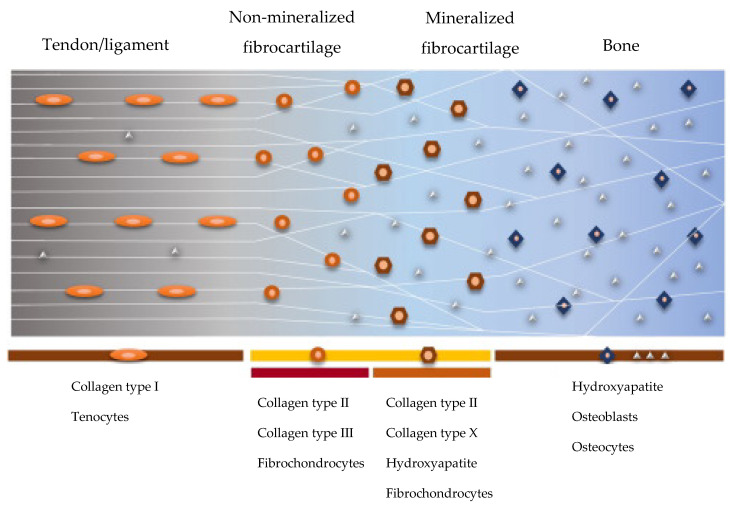
Schematic representation of the structure and composition of the direct enthesis. Adapted with permission from Ref. [3]. Copyright 2022, University of Pavia.

Where the tissue is more subjected to recurring loading forces, such as in the cruciate ligament, rotator cuff, and Achilles tendon, the enthesis tissue is directly connected to the bone, which presents discontinuous periosteum in the fixation site. The direct enthesis includes four gradient zones (Figure 1): first, the tendon zone, is characterized by aligned fibers of type I collagen; the second mainly consists of fibrocartilage (collagen type II and III); the third zone is characterized by mineralized fibrocartilage (type II and X collagen and hydroxyapatite in gradient); and lastly, the bone (type I collagen and minerals). The TBI between fibrocartilaginous and mineralized fibrocartilaginous tissue creates the stiffness gradient throughout the soft tendon and the hard bone during joint movement. This connection is poorly vascularized and is frequently affected by scratches or complete lacerations, since it is often submitted to high mechanical stress [1,2].

Nowadays, orthopedic treatment of the native tendon, which is surgically inserted into the bone, is not able to recreate the native direct enthesis, leading to the formation of scar tissue covering the periosteum surface, with inferior biomechanical properties [4]. The development of tissue engineered bio-mimetic scaffolds able to link the tendon–bone gap, and support the mechanical loading, has gained attention in the recent years as a promising approach for the treatment of TBI injuries [4]. However, this scaffold alone is insufficient for controlling all the healing phases of this particular tissue.

Given these premises, the aim of this study was the design and development of a topographical and compositional gradient tubular scaffold based on pullulan (PU) and chitosan (CH), to be implanted at TBI site. CH was selected as it is a well-known polymer and characterized by remarkable bioadhesion, biocompatibility, biodegradability, antimicrobial activity, and wound healing properties [5,6].

However, CH is not easily spinnable and for this reason it was combined with PU, an easily spinnable polysaccharide having numerous food, pharmaceutical, and biomedical applications [6,7]; thus, allowing a one step process that is easy to set up.

The tubular shape of the scaffold was designed to fulfill two functions: as a delivery system for biological modulators, and as a support for native cell growth and mechanical reinforcement.

The tubular scaffold was manufactured by means of electrospinning. The nanofibers were collected with two different conformations: one part aligned (namely: A), and the other one random (namely: R), to obtain a topographical and inorganic nanomaterial gradient that should allow a gradient mimicking that at the enthesis site. Hydroxyapatite (HAP) nanoparticles were loaded into random fibers, creating a doped scaffold part intended for the bone region (R-HAP), while aligned undoped fibers (blank, B) directed along the tube length formed the opposite extremity of the scaffold, intended for the tendon part (A-B). Finally, chondroitin sulfate (CS) was selected as a biomimetic niche to be loaded into the tubular scaffold void, to effectively deliver CS, as a biological modulator, to the injured site. In vitro evaluation was performed using human adipose-mesenchymal stem cells (hASCs), as they are considered an appropriate model for studying the osteo/teno-genic properties of the scaffold [8,9].

## 2. Experimental Section

### 2.1. Materials

Chitosan (CH), deacetylation degree 98%, MW 251000 Da, (ChitoClear, Siiiglufjordur-Iceland); pullulan (PL), food grade (Hayashibara, Okayama-Japan); citric acid (CA) (Carlo Erba Reagents, Italy); hydroxyapatite (HAP), nanopowder < 200 nm particle size, ≥97% synthetic (Sigma-Aldrich, USA); acetic acid (AA) (Carlo Erba Reagents, Italy); chondroitin sodium sulfate (CS) (β-1,4-linked D-glucuronic acid and β-1,3-linked N-acetyl galactosamine) bovine 100 EP, low MW 14 kDa, mixture of chondroitin A (chondroitin 4 sulfate), and chondroitin C (chondroitin 6 sulfate) (Bioiberica, Italy).

### 2.2. Methods

#### 2.2.1. Preparation of the Polymeric Blends

First, 20% (*w*/*w*) PL was dissolved in water, while 5% (*w*/*w*) CH and 5% (*w*/*w*) CA were both solubilized in acetic acid:water (90:10), under magnetic stirring, at room temperature [9,10].

The polymeric blend was prepared by mixing the two polymeric solutions previously prepared in a 1:1 weight ratio, to obtain the undoped polymer solution (A-B). The 0.1 HAP solution (R-HAP) was prepared by adding HAP nanoparticles to the final polymeric blend, 1 h before electrospinning, to obtain a 0.1% (*w*/*w*) final HAP doping.

#### 2.2.2. Preparation of the Tubular Scaffold

The scaffold was obtained using an electrospinning apparatus (STKIT-40, Linari Engineering, I), equipped with a high-voltage power supply (Razel R99-E 40, kV), a 10 cc syringe with inox 21 G needle and a volumetric pump (Razel R99-E). This process was carried out at atmospheric pressure, in a 25–35 °C temperature range, and 25–35% relative humidity, with a spinneret size of 0.7–0.8 × 20 mm. The distance between the tip of the needle and the collector was 15 cm, with 0.397 cc/h continuous flux and 20–22 kV voltage.

The nanofibers were collected using a cylindrical inox-steel rotating drum (3 mm diameter × 15 cm length, Easy Drum).

The tubular scaffold was prepared using two sequential collections: at first, HAP doped solution (R-HAP) was electrospun, and then the nanofibers were collected under slow clockwise rotation at 2000 rpm for 1 h, to form a random HAP doped structure; subsequently, one end was covered and the undoped polymer solution (A-B) was electrospun at 3000 rpm clockwise rotation, adding a simultaneous 60 oscillations/min longitudinal movement, to obtain the longitudinal disposition fibers (alignment).

Finally, the resulting tubular scaffold was crosslinked using a dry heating treatment at 150 °C for 1 h. This heating process is also reported as being appropriate for scaffold sterilization [10,11,12].

#### 2.2.3. Structural Characterization

The scaffold morphology was characterized by means of SEM analysis after carbon sputtering (Tescan, Mira3XMU, Brno, Czech Republich).

The different regions (A-B, R-HAP and the interface (middle region)) of the scaffolds were analyzed in the native state (after the manufacturing), and after 6 days of hydration in PBS (pH 7.4 phosphate buffer solution, Sigma Aldrich, Milan, Italy). In this last case, before sputtering, the scaffolds were dehydrated using ethanol gradient (50–100%). The nanofiber dimensions were measured using image analysis from the SEM images.

The HAP distribution in the tubular scaffold was evaluated using Alizarin Red (AR) staining. For this purpose, 2% *w*/*v* AR solution (Merk, Germany) in distilled water at 4.1 pH was prepared, and the scaffolds (4 cm length, 3 segments along the tubular scaffold) were dipped for 5–10 min, until the reaction was visible microscopically. The scaffold portions rich in calcium appeared orange-red. The images were recorded using an optical microscope (Leica Microsystem, Portugal).

The microstructure was then analyzed using X-ray micro-computed tomography (micro-CT) and a high-resolution X-ray microtomography system (Skyscan 1272, Bruker, Billerica, MA, USA). The acquisition of X-ray images was performed with a pixel size of 10 μm, a rotation step of 0.4° over 360°, and a smoothing averaging of every three images. The X-ray source was fixed at 50 kV voltage and 200 μA current. After the acquisition, the gray-scale images were reconstructed using NRecon software (version 1.7.1.0, Bruker, Billerica, MA, USA). Moreover, the tubular scaffold was vertically aligned for longitudinal analysis of the scaffold thickness using DataViewer software (version 1.5.3.6, Bruker, Billerica, MA, USA). Qualitative visualization of the 3D morphology and the different phases of the polymeric matrix and HAP nanomaterial were performed using CT-Vox software (version 3.3.0, Bruker, Billerica, MA, USA). Finally, a quantitative analysis was carried out, after converting the regions of interest into binary images using a dynamic threshold (30–255—polymeric phase; 80–255 ceramic phase). The binary images were used for morphometric examination (CT Analyzer v1.12.0.0, SkyScan, Kontich, Belgium) [7].

Structural analysis on the nanoscale was performed using small angle x ray scattering, SAXS, at the European Synchrotron Radiation Facility (ESRF, Grenoble, France), carrying out experiments with the ID02 beamline (Del Favero E., Bianchi, E., Ruggeri, M; structural characterization of insoluble hydrophilic scaffolds for wound healing (Data set); European Synchrotron Radiation Facility. https://doi.org/10.15151/ESRF-ES-585935736, accessed on 22 August 2022). Scaffolds were inserted in 2-mm polycarbonate capillaries (ENKI, Italy) and fully hydrated with water. The scattered radiation was collected at two different sample-to-detector distances (1 m and 10 m), to investigate a wide range of q = 4πsen(θ/2)/λ, with θ being the scattering angle and λ = 0.1 nm the x-ray wavelength. The intensity spectra as a function of q (0.006 < q < 7.5 nm^−1^) gave information on the mesoscale arrangement of the fibers (hundreds of nanometers) and on the local arrangement of polymer chains (nanometer length-scale). Measurements were repeated on different samples, to check for reproducibility.

#### 2.2.4. Biocompatibility

Cytocompatibility, adhesion, and proliferation assays were carried out using hASCs cells provided by ZenBio (Durham, NC, USA). The hASCs were cultured in basal-α-MEM medium based on 1% of minimum essential medium (MEM, ZenBio, Durham, NS, USA), supplemented with 10% FBS (fetal bovine serum, Euroclone, Pero (MI), Italy), 0.22% bicarbonate, and 1% *v*/*v* penicillin-streptomycin-amphotericin (pen/strep/ampho 100×, Euroclone, Pero (MI), Italy). The cells were grown in an incubator (CO_2_ Incubator, PBI International, Milano, Italy) at 37 °C with 5% CO_2_ and 95% relative humidity (RU).

Before the seeding, the scaffold diskettes were placed in a 96-well plate and sterilized under UV irradiation for 20 min. The tubular scaffolds were cut at both the A-B and R-HAP parts to produce 0.2 cm^2^ area diskettes (diameter of 5 mm, 0.2 mm thickness).

For the preliminary biocompatibility assay, scaffolds were dipped in 100 µL MEM for 48 h, and the extract was put in contact with hASC substrates (20 × 10^3^/well (96 well plate)) grown for 24 h.

For the adhesion, proliferation, and differentiation assay, hASCs were seeded onto the diskette surfaces, with a density of 20 × 10^3^/well (96 well plate). Then, 100 μL cell suspension in basal medium was carefully dropped onto the scaffold diskettes and left in an incubator (37 °C, 5% CO_2_) for 30 min, and culture medium was added up to 200 µL. Analogously, the experiment was performed in the presence of CS. For this purpose 3% *w*/*w* CS solution was prepared in full basal-α-mem medium (α-MEM) for hASCs. The polymer solution was left overnight for hydration and filtered in a sterilizing filter with a 0.22 µm pore size, and this was used as the basolateral medium.

After 6, 14 days, and 21 days, an Alamar Blue test (AlamarBlue HS cell viability reagent, Invitrogen, Thermo Fisher, Monza, Italy) was performed to evaluate the metabolic activity (viability) of the cells. Briefly, the metabolic activity was studied as the fluorescence value obtained by the REDOX reaction of the Alamar Blue (Alamar Blue, Bio-Rad) from resazurin (Alamar Blue) to resorufin (pink fluorescent dye). At a predetermined time, the scaffold diskettes were washed with D-PBS and were treated with 10% (*v*/*v*) Alamar blue in α-basal medium. After 3 h of dark incubation (37 °C, 5% CO_2_), they were collected and transferred to new wells. Alamar Blue fluorescence was recorded using a microplate reader (Microplate Reader Biotek, Synergy/HT, Fisher Scientific, Rodano (MI), Italy) with λex = 530 nm and λem = 590 nm. Cytocompatibility was estimated as the ratio between the fluorescence intensities collected for the samples and the standard growth condition (growth medium, GM).

#### 2.2.5. Immunofluorescence Analysis

Cells grown on the scaffolds (A-B or R-HAP) were fixed using a 3% (*v*/*v*) glutaraldehyde solution in PBS (Sigma-Aldrich, Milano, Italy) for 2 h at room temperature. The substrates were then washed three times with PBS. Extracellular matrices were stained using anti-collagen I rabbit polyclonal antibody (Thermofisher, Monza, Italy; 100 µL/sample at 10 µg/mL in PBS) to immuno-label collagen I produced from hASCs differentiated in TEN-1 (A-B diskettes) or bone sialoprotein antibody (Thermofisher, Monza, Italy; 100 µL/sample at 1 µg/mL in PBS) to immuno-label sialoprotein produced from hASCs differentiated in osteoblasts (R-HP diskettes) (24 h contact time at 4 °C). Each primary antibody was stained with ATTO 488 goat anti rabbit IgG (Sigma Aldrich, Milano, Italy), as secondary antibody (green). Cellular cytoskeleton was stained with TRICT-phalloidin (Sigma-Aldrich, Milano, Italy; 50 µL/sample at 50 µg/mL in PBS) for 40 min, in the dark.

Then, each substrate was washed twice, and cell nuclei were stained with Hoechst 33258 (Sigma-Aldrich, Milano, Italy; 50 µL/sample at 1:10,000 in PBS) for 10 min in the dark. Scaffold diskettes were placed onto microscope slides and imaged using a confocal laser scanning microscope (CLSM, Leica TCS SP2, Leica Microsystems, Buccinasco (MI), Italy) with (a) λex = 346 nm and λem = 460 nm for Hoechst 33258; (b) λex = 540 nm and λem = 565 nm for TRICT phalloidin; and (c) λex = 501 nm and λem = 523 nm for ATTO 488 goat anti rabbit IgG. The acquired images were processed with software (Leica Microsystem, Buccinasco (MI), Italy).

#### 2.2.6. Statistical Analysis

Statistical analysis was performed using a post hoc Tukey HSD test calculator. One-way ANOVA followed by Scheffé, Bonferroni, and Holm methods were considered. For the comparison of the two groups, statistical significance was determined by using a two-tailed Student’s *t*-test method. A *p*-value ≤ 0.05 was considered statistically significant.

## 3. Results and Discussion

### 3.1. Structural Characterizations

**Figure 2:**
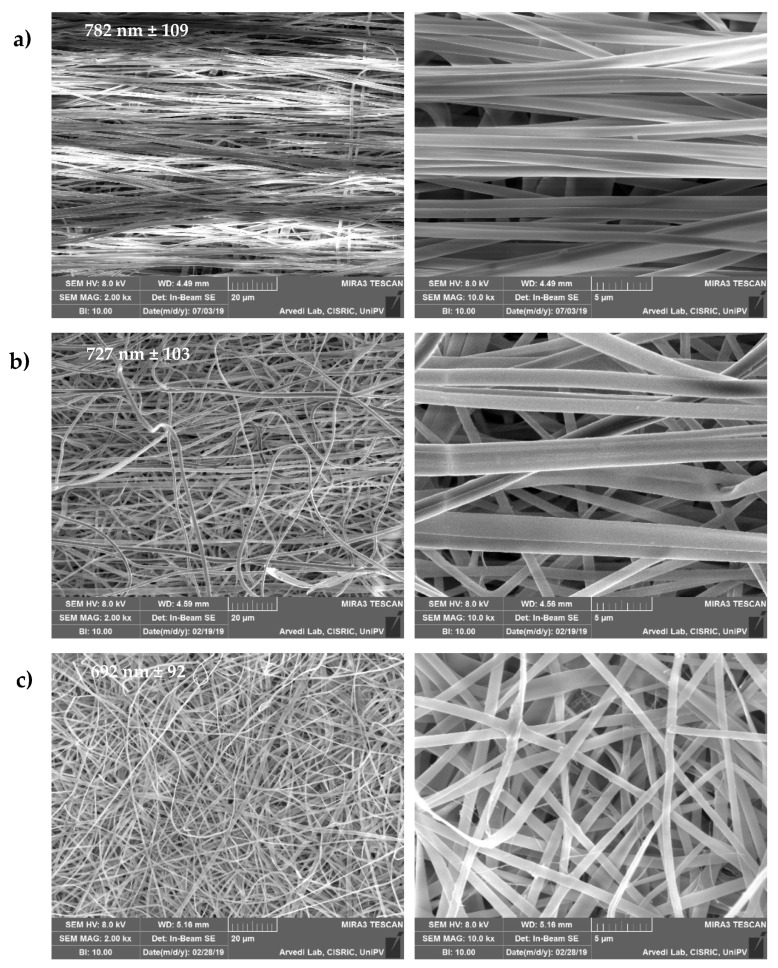
SEM images (2 and 10 kx, left and right panels, respectively) of the scaffold: (**a**) A-B, aligned undoped part; (**b**) interface region between the A-B and R-HAP parts; (**c**) R-HAP, random HAP doped nanofibers. In the images, the nanofiber sizes are reported (mean values ± sd; *n* = 8).

The topographical and compositional gradient of the assembled tubular scaffold was prepared by means of electrospinning with sequential manufacturing, to create the 3D-structured end parts; one randomly organized and HAP doped, with a high affinity for the bone tissue, and the other one having aligned nanofibers, without nanomaterial doping, and able to simulate the tendon organization. The tubular structure was designed to load active components into the inner cavity of the device, to enhance tissue repair, as in the case of platelet lysate [13]. Figure 2 provides SEM images of the 3D structure.

The fibers size was influenced by the HAP doping and the alignment of the fibers: the HAP doped nanofibers were characterized as having a 692 nm (±92 nm) diameter, while the undoped fibers had larger diameters of 782 nm (±109 nm). Moreover, in the interface region, the fibers had intermediate dimensions of 727 nm (±103 nm). The surface of the single nanofibers forming the scaffold appeared smooth, both for the random HAP doped and the undoped aligned fibers. The average nanofiber diameters, always in the nanometric range, were slightly larger in the aligned region with respect to the HAP doped region, although the differences were not significant.

**Figure 3:**
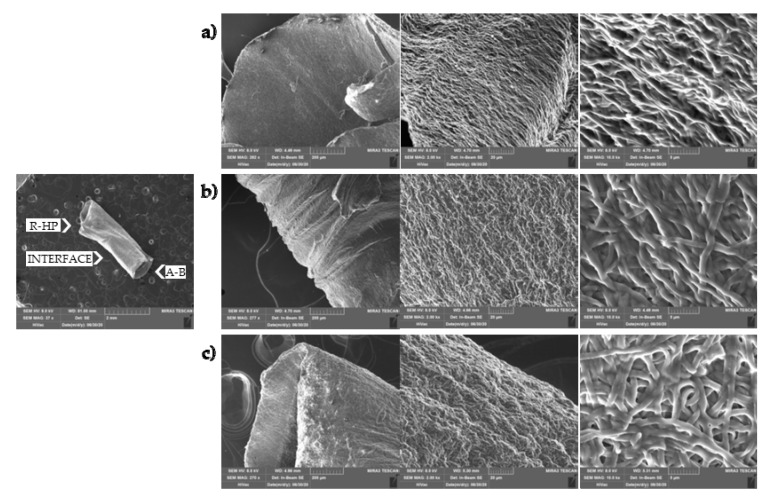
SEM (0.02, 0.2, 2, and 10 kx, left, middle, and right panels, respectively) images of the tubular scaffold after hydration in PBS for 6 days: (**a**) A-B part; (**b**) interface region between the A-B and R-HAP parts; (**c**) R-HAP part.

Figure 3 shows images of the tubular scaffold after 6 days of hydration. The hydration did not alter the tubular structure and the inner void is clearly visible at low magnification. At higher magnifications, it is possible to note that the nanofibrous structure has been preserved, as well as the random or aligned organization of the nanofibers. After hydration, all the regions were characterized by nanofibers of similar dimensions: HAP doped nanofibers had a 786 nm (±57 nm) diameter, the undoped fibers were 794 nm (±45 nm), and in the interface region the fibers were 798 nm (±66 nm). Furthermore, in the HAP doped part and in the interface region, the nanofibers appear swollen and they were increased by 12% and 9%, respectively.

**Figure 4:**
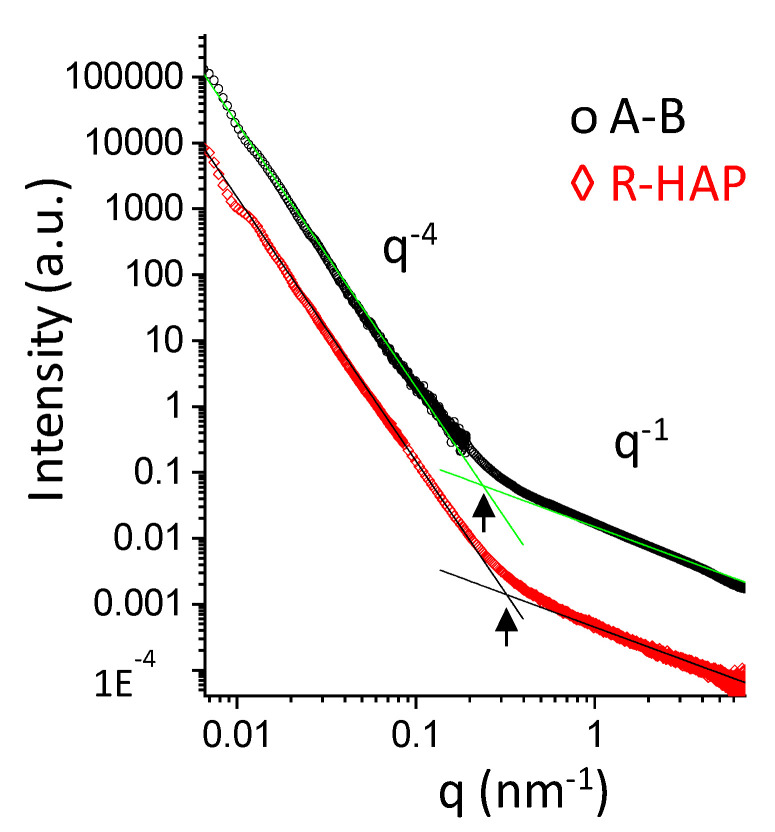
SAXS spectra of A-B and R-HAP regions. Straight lines represent the two different power-low of the scattered intensity decay: I(q) ÷ q^−4^ in the low-q range, I(q) ÷ q^−1^ for q > 2 × 10^−1^ nm^−1^ in the A-B region, and at q > 3 × 10^−1^ nm^−1^ in the R-HAP region.

The nanoscale structure of tubular scaffolds was investigated sing small angle x-ray scattering (SAXS) in wet state, to simulate an implant. The SAXS spectra of the A-B and R-HAP regions are reported in Figure 4. In the low-q range, giving structural information in a length-scale of hundreds of nanometers, the intensity decays are similar and follow a q^−4^-slope typical of objects with a smooth surface. In the high-q range, the intensity profile of both systems deviates from the q^−4^ steep decay. This behavior, in the length-scale of nanometers, reveals the local arrangement of polymer chains belonging to the fibers. The q^−1^-slope is typical of elongated chains protruding from the surface of the fibers. Interestingly, a change of the slope is observed at q ≅ 2 × 10^−1^ nm^−1^ in the A-B region, and at q ≅ 3 × 10^−1^ nm^−1^ in the R-HAP region, corresponding to the typical longer length of ≅30 nm for A-B elongated chains and to a shorter one (≅20 nm) for the chains in the R-HAP region.

**Figure 5:**
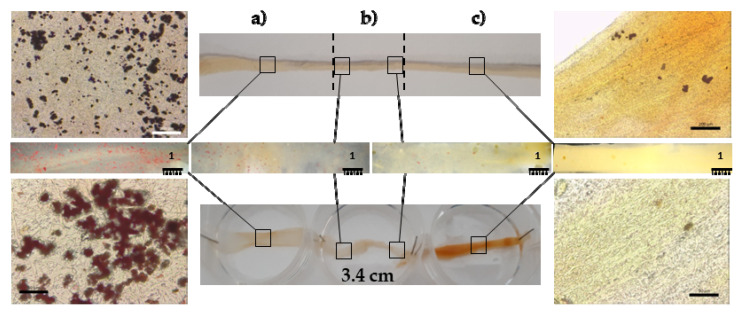
Optical images of Alizarin Red staining of the tubular scaffold: (**a**) R-HAP part; (**b**) interface; (**c**) A-B part.

The HAP gradient is evident in Figure 5, where the inorganic nanomaterial is stained using Alizarin Red. Round spots, conceivably related to HAP aggregates in the structure, are visible in the random HAP doped part (Figure 5a), resulting in an organization similar to bone tissue. These greatly decrease in the interface region, while in the undoped aligned part (Figure 5c), similar to the tendon organization, only rare red aggregates are visible. This should mimic the gradient of the inorganic matrix in the native tissue at the bone–tendon interface, similarly to the bone–enthesis–tendon organization.

Considering these findings, the inner cavity allows the extemporaneous loading of chondroitin sulphate (CS) and the implanting of the loaded system during the surgical intervention. This should allow bridging the tendon lesions and supporting the repairing process and the controlled and prolonged release of CS, to support tissue repair in the post-surgery period.

**Figure 6:**
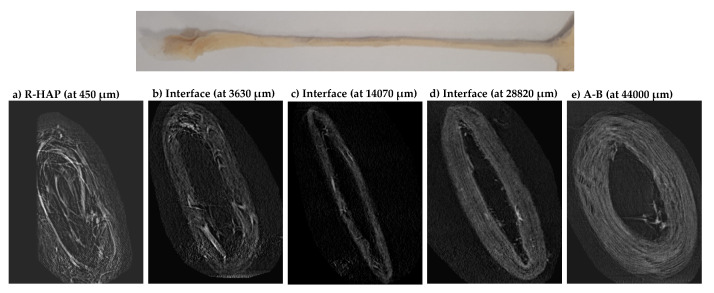
Micro CT images of the cross-sections acquired along the length of tubular scaffold from (**a**) the bone part (R-HAP), to (**e**) the tendon part (A-B), throughout (**b**), (**c**), and (**d**) the interface area.

The micro CT analysis (Figure 6) confirmed the presence of a morphological and compositional gradient along the tubular scaffold, without interruptions. In particular, the 2D micro CT images show the scaffold cross-section along the tubular length. It is possible to recognize the HAP doped part, the interfacial region, and the undoped part. The analysis confirmed the random nanofiber assembly of the R-HAP part and the alignment of the undoped part. The initial inner cavity (3 mm in diameter) seems directly related to the alignment and shows a reduced void in the R-HAP region. The inner cavity is preserved from the aligned organization of the nanofibers, while in the random portion the 3D organizations of the nanofibers and the synergic effect of the highly hydrophilic HAP seem to contribute to increasinf the structures flexibility, and the inner cavity is more prone to collapse.

In a previous work [13], the HAP doping and the random or aligned organization proved to possess different mechanical properties: the aligned part was more rigid (in the elastic regime, higher Young’s modulus) than their random counterparts, and able to withstand a higher stress and larger deformation before breaking, and on the other hand, it appears that the moderate HAP doping in the random part was significantly the most beneficial factor for the mechanical performance of the scaffolds, strengthening their structure [13]. Furthermore, both the parts were characterized by mechanical properties, both elasticity and force-at-break, similar to those of native tendons, as they typically have an ultimate tensile strength which ranges from 5 to 100 MPa, with a strain of failure 10–15% and a Young’s modulus from 20 to 1200 MPa [13].

### 3.2. Biopharmaceutical Characterization

**Figure 7:**
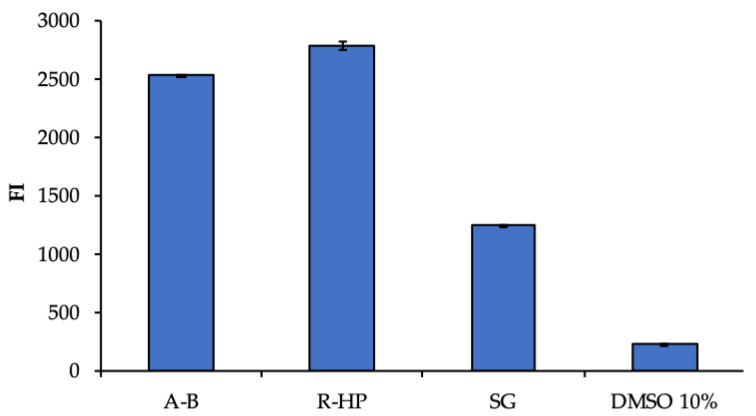
Viability (FI, fluorescent intensity) of human adipose stem cells (hASCs) grown for 3 days in the presence of extracts from the two parts of the scaffold (R-HAP and A-B). GM (growth medium) and 10% DMSO were used as positive and negative controls, respectively (mean values ± sd; *n* = 8). ANOVA one-way; Scheffé test: A-B vs. R-HAP: *p* < 0.01; A-B vs. GM: *p* < 0.01; A-B vs. DMSO 10%: *p* < 0.01; R-HAP vs. GM: *p* < 0.01; R-HAP vs. DMSO 10%: *p* < 0.01; GM vs. DMSO 10%: *p* < 0.01.

A preliminary biocompatibility analysis was performed, to evaluate the cytotoxicity of scaffold components towards hASCs. Figure 7 reports the hASC viability (fluorescent intensity) after contact with the extracts obtained from the different portions (R-HAP and A-B) of the scaffold, in comparison with the standard growth conditions (GM), as positive control, and 10% DMSO, as cytotoxic reference. The significantly higher cell viability obtained after the contact with the extracts from the random HAP doped part and the undoped aligned part suggests that the whole scaffold was characterized by a good biocompatibility, and the scaffold extracts were able to enhance the hASC proliferation better than GM. As expected, DMSO presented a high cytotoxicity. This is supported by the findings from other authors that emphasized the use of chitosan and pullulan to enhance the hASC proliferation in systems intended for implant [14,15]. Moreover, the HAP nanomaterial is also recognized as able to favor hASCs differentiation and proliferation of culture [16]. This assay was performed as a preliminary analysis, to evaluate the eventual release of toxic components from the scaffold.

**Figure 8:**
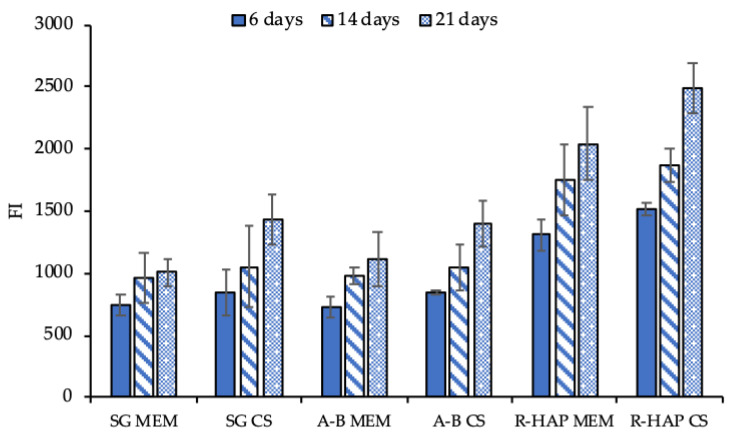
Viability (FI, fluorescence intensity) of hASCs grown onto the A-B and R-HAP parts of the tubular scaffold after 6, 14, and 21 days in culture with MEM (growth medium) and MEM supplemented with 3% *w*/*w* CS (mean values ± sd; *n* = 8). ANOVA one-way; Scheffé test (*p* ≤ 0.05): 6 days: SG MEM vs. R-HAP MEM: *p* < 0.01; SG MEM vs. R-HAP CS: *p* < 0.01; SG CS vs. R-HAP MEM: *p* < 0.01; SG CS vs. R-HAP CS: *p* < 0.01; A-B MEM vs. R-HAP MEM: *p* < 0.01; A-B MEM vs. R-HAP CS: *p* < 0.01; A-B CS vs. R-HAP MEM: *p* < 0.01; A-B CS vs. R-HAP CS: *p* < 0.01; 14 days: SG MEM vs. R-HAP MEM: *p* < 0.01; SG MEM vs. R-HAP CS: *p* < 0.01; SG CS vs. R-HAP MEM: *p* = 0.0253; SG CS vs. R-HAP CS: *p* < 0.01; A-B MEM vs. R-HAP MEM: *p* < 0.01; A-B MEM vs. R-HAP CS: *p* < 0.01; A-B CS vs. R-HAP MEM: *p* = 0.0494; A-B CS vs. R-HAP CS: *p* < 0.01; 21 days: SG MEM vs. R-HAP MEM: *p* < 0.01; SG MEM vs. R-HAP CS: *p* < 0.01; SG CS vs. R-HAP MEM: *p* < 0.01; SG CS vs. R-HAP CS: *p* < 0.01; A-B MEM vs. R-HAP MEM: *p* < 0.01; A-B MEM vs. R-HAP CS: *p* < 0.01; A-B CS vs. R-HAP MEM: *p* < 0.01; A-B CS vs. R-HAP CS: *p* < 0.01; A-B MEM 6 days vs. A-B MEM 14 days: *p* = 0.0121; A-B MEM 6 days vs. A-B MEM 21 days: *p* < 0.01; A-B CS 6 days vs. A-B CS 21 days: *p* < 0.01; A-B CS 14 days vs. A-B CS 21 days: *p* < 0.01; R-HAP MEM 6 days vs. R-HAP MEM 14 days: *p* = 0.0412; R-HAP MEM 6 days vs. R-HAP MEM 21 days: *p* < 0.01; R-HAP CS 6 days vs. R-HAP CS 14 days: *p* < 0.01; R-HAP CS 6 days vs. R-HAP CS 21 days: *p* < 0.01; R-HAP CS 14 days vs. R-HAP CS 21 days: *p* < 0.01.

**Figure 9:**
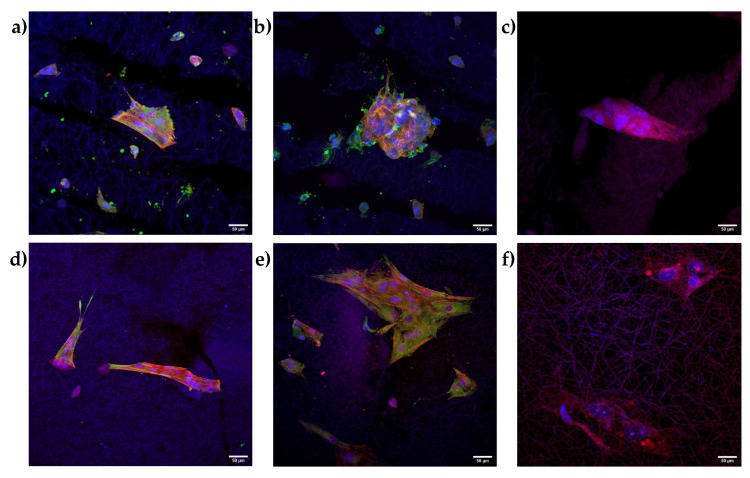
CLSM images of hASCs grown onto the A-B (**a**–**c**), (**upper row**) and R-HAP (**d**–**f**), (**lower row**), with (**b**,**e**) and without (**a**,**d**) CS in the medium, after 21 days. Actin stained in red, nuclei stained in blue, collagen I (**a**,**b**), (**upper row**) stained in green, sialoprotein (**d**,**e**), (**lower row**) stained in green. The right column (**c**,**f**) shows the negative control with A-B scaffolds stained with sialoprotein (**c**), (**upper row**) and R-HAP scaffolds stained with collagen I (**f**), (**lower row**) (magnifications: 20×).

To support the intended use of the scaffold as a tendon substitute to bridge the tendon to bone structure, and to enhance enthesis repair, the adhesion, proliferation, and differentiation of hASC onto the A-B and R-HAP parts of the scaffold were assessed after 6, 14, and 21 days (Figure 8 and Figure 9). The investigation was performed in standard growth conditions, and also by supplementing the growth medium with 3% *w*/*w* CS, to simulate the tubular structure loading upon implant.

As shown in Figure 8, CS significantly increased the stem cell proliferation, both for the control (SG) and the cells grown onto the scaffold surface. Moreover, both A-B and R-HAP were proven to act as an effective support to sustain cell proliferation for up to 21 days. This suggests that CS significantly contributes to the hASC homing, resulting in a suitable niche for proliferation and differentiation, and enabling the carrier to load and control the delivery of active components post-surgery, thanks to the unique tubular 3D structure.

The hASC differentiation was evaluated at the end of the culture, after 21 days, by means of CLSM analysis; in particular, the cell morphology and extracellular matrix production, either a tendon-like or osteo-like matrix, were considered (Figure 9).

The most relevant proteins related to tendons are collagen, tenascin, decorin, tenomodulin, and versican, while scleraxis (SCXA) is found in the early stage of the progenitor cell differentiation in tendons and is employed during the embryonic development, to form tendons and blood vessels. It also remains highly expressed in all adult connective tissues and is thought to play a role in tendon–bone attachment [17,18].

SCXA, a basic helix-loop-helix transcription factor, has a fundamental role in mesenchymal stem cell differentiation in tenocytes. Despite the early production of this factor in hASC differentiation, it is secreted both by progenitor cells and differentiated ones. For this reason, collagen I has been detected as a marker of hASC differentiation in tenocytes, being a tendon ECM component. Therefore, the collagen I production from hASCs cultured both with and without CS supplementation onto the A-B scaffolds was evaluated. Moreover, the production of sialoprotein from hASCs cultured both with and without CS supplementation onto the R-HAP scaffolds was also evaluated, to understand their potential to mimic bone tissue. The CLSM images suggest a partially anisotropic behavior of the hASCs. The green signal of the sialoprotein is clearly visible in the proximity of the cells grown onto R-HAP scaffolds (Figure 9d,e), indicating the production of sialoprotein. The collagen I proteins marked in green were also localized in the proximity of hASC grown onto A-B scaffold (Figure 9a,b). On the other hand, when the R-HAP scaffold was stained with collagen I (Figure 9f) and the A-B scaffold was stained with sialoprotein (Figure 9c), no green signal was visible, indicating that the substrate leads to a unique differentiation, either in tenocytes for A-B (aligned blank, not HAP doped) or in osteoblasts for R-HAP (random, HAP doped).

In vivo, the crucial mechanisms leading to bone formation involve the chloride channels (ClCs) at the apical membrane of osteoblasts. The ClCs uptake protons coming from the hydroxyapatite synthesis (mineral precipitation in the bone) and transfer them through the basolateral membranes in the extracellular fluids. This ionic equilibrium supports the complete conversion of phosphate and calcium into hydroxyapatite crystals. Given this, it has been proven that calcium phosphate ceramics induce stem cell differentiation towards osteoblasts [19].

Overall, the preliminarily results suggested that the A-B part provides a suitable environment for the promotion of newly synthetized tendon ECM, while the R-HAP part produced a mineralized bone-like matrix deposition. Moreover, the CS presence increases the cell proliferation and, consequently, both the synthesis of ECM of the tendon and osteoblast (collagen I and sialoprotein, respectively).

## 4. Conclusions

A tubular scaffold based on pullulan/chitosan, designed with a tendon- and bone-like parts, and to be implanted in the TBI was developed. The scaffold showed a topography gradient of electrospun nanofibers, from random (R-HAP) deposition to aligned (A-B), in the direction along the tubular length, for the bone and tendon parts, respectively. The HAP doping further increased the stem cell differentiation to osteoblasts. The continuous structure with an interface region, where the fiber alignment and HAP nanomaterial content gradually changed from one part to the other, seemed effective in inducing hASC differentiation towards tendon-like cells or osteoblasts, as a function of the scaffolds morphology and composition. In particular, the in vitro evaluation suggested that osteogenic and tenogenic differentiation was greatly supported by chondroitin sulfate.

Although in vivo experiments are needed, this tubular scaffold is a promising device as a multi-functional surgical support in enthesis tissue reparation.

## Data Availability

Data available on request.
